# Safe and successful teclistamab treatment in very elderly multiple myeloma (MM) patients: a case report and experience from a total of three octogenarians

**DOI:** 10.1007/s00277-023-05451-8

**Published:** 2023-09-15

**Authors:** Martin Philipp Dieterle, Gila Mostufi-Zadeh-Haghighi, Jan Wilhelm Kus, Christopher Wippel, Zacharias Brugger, Cornelius Miething, Ralph Wäsch, Monika Engelhardt

**Affiliations:** 1 Department of Hematology, Oncology and Stem Cell Transplantation, Medical Center - University of Freiburg, Faculty of Medicine, University of Freiburg, Freiburg, Germany; 2 Center for Dental Medicine, Division of Oral Biotechnology, Medical Center—University of Freiburg, Faculty of Medicine, University of Freiburg, Hugstetterstr. 55, 79106 Freiburg, Germany

Dear Editor,

We here report on a 82-year-old patient with relapsed/refractory multiple myeloma (RRMM), who was successfully treated with the T cell engaging bispecific antibody teclistamab targeting B cell maturation antigen (BCMA) [1–4]. Our patient was newly diagnosed with lambda (λ) light chain (LC) MM in July 2021 at age 80 and initially presented with cervical pain. A whole-body computed tomography (WB-CT) scan and laboratory investigations revealed multiple osteolytic lesions, anemia (13.2g/dL) and impaired kidney function (serum creatinine 1.21mg/dL; GFR 58mL/min/1.73qm) representing symptomatic MM fulfilling 3/4 C**RAB** criteria (hypercalcemia, renal dysfunction, anemia, bone involvement). Bone marrow biopsy confirmed a substantial plasma cell infiltration of 80% and unfavorable cytogenetics (t(11;14); monosomy 13; CKS1B gene amplification in 1q21), the ISS/R-ISS were I and II, respectively, and his λ-serum free light chains (FSLCs) were elevated at 236mg/L. His revised myeloma comorbidity index (R-MCI) was 5/9 (=intermediate-fit), reflecting relevant comorbidities including NSTEMI, multivessel coronary artery disease, and severe aortic valve stenosis [5, 6].

**Fig. 1:**
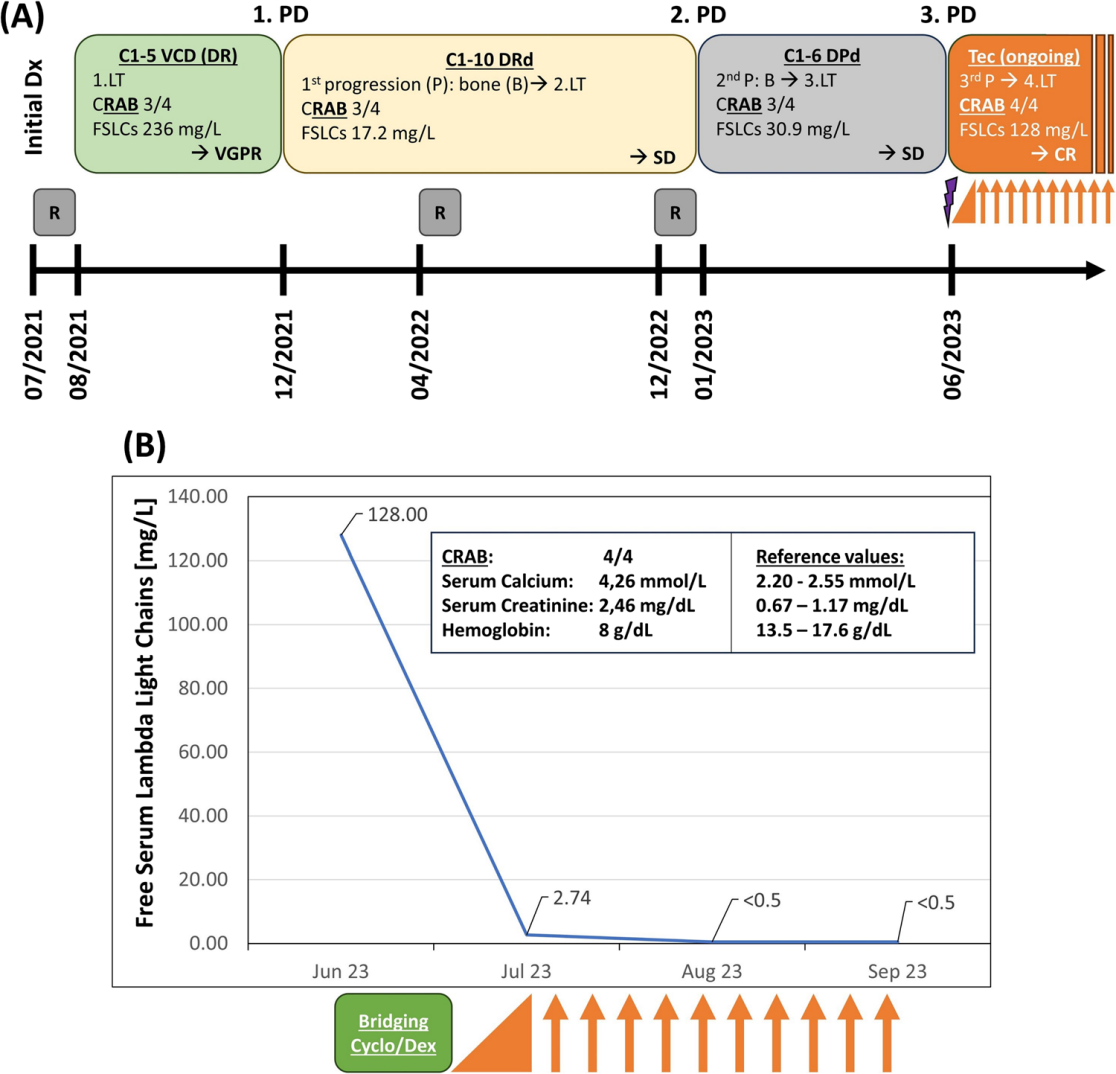
**A** Treatment regimens in our patient over a course of 2 years and **B** free serum lambda light chain (FSLC) decline upon response to teclistamab treatment. **A** At initial diagnosis (Dx) in 07/2023, the 80-year-old patient presented with λ-serum free light chains (FSLCs) of 236mg/L and fulfilled 3 out of 4 CRAB criteria (fulfilled ones depicted bold and underlined). After initial radiation therapy (R), he received five cycles (C) of VCD (bortezomib/cyclophosphamide/ dexamethasone) as a first line therapy (LT) leading to a very good partial response (VGPR). Upon bone (B) progression (P) (=first progressive disease, 1.PD), 10 C of DRd (Daratumumab/lenalidomide/dexamethasone) were administered. Stable disease (SD) was achieved. Due to again progressive bone disease (2.PD), the therapy was switched to DPd (Daratumumab/pomalidomide/dexamethasone) for six C. This led to SD again. **B** In June 2023, the patient presented with 4/4 CRAB criteria and FSLCs of 128mg/L (=3.PD). Due to his substantial disease burden, a bridging therapy with 200mg/day cyclophosphamide (Cyclo) and dexamethasone (Dex) 20mg/day (purple arrow in A/green box in B) was applied for 5 days until teclistamab (Tec) was followed. Tec was given as part of a compassionate use program. Ramp-up doses of teclistamab (orange triangle; corresponds to C1 with the following three doses: day 1: 0.06mg/kg; day 3: 0.3mg/kg; day 5: 1.5mg/kg) were administered. Subsequently, the bispecific antibody was administered subcutaneously once weekly (1.5 mg/kg) in the outpatient setting (orange arrows; representing ongoing Cs) and is well tolerated. Upon Tec response, FSLCs impressively dropped to <0.5mg/L. Serum and urine became immunofixation negative (CR; see further details in Supplementary Table 1)

During first-line therapy (1.LT), the patient received radiation therapy of symptomatic bone lesions and five cycles of bortezomib-cyclophosphamide-dexamethasone (VCD; cyclophosphamide: 750mg absolute dose day (d) 1 i.v.; dexamethasone 20mg; bortezomib 1.3mg/m^2^ s.c. d1,8,15), achieving good 1.LT tolerance and very good partial response (VGPR; Fig. 1A). Due to bone progression in December 2021, a 2.LT with daratumumab-lenalidomide-dexamethasone (DRd) was administered for a total of 10 cycles, achieving serological stable disease (SD), painless, constant bone lesions, and good quality of life (QoL). Due to WB-CT confirmed bone progression with exacerbating pain in January 2023, daratumumab-pomalidomide-dexamethasone (DPd) was given as 3.LT for 6 cycles. However, 4/4 CRAB symptoms with hypercalcemia, impaired renal function, anemia, and bone pain reoccurred, verified by increased λ-SFLCs (128mg/L; Fig. 1A, B). After having received three prior lines of therapy, including a proteasome inhibitor (PI), two immunomodulatory drugs (IMiDs), and an anti-CD38-antibody, the patient presented with triple-refractory RRMM (Fig. 1B). His general condition had deteriorated (Eastern Cooperative Oncology Group [ECOG] 2; R-MCI: 7/9=frail), accompanied by weight loss and neutropenia. Since the patient, his family, and our treatment team remained supportive of further anti-myeloma treatment, the patient was enrolled in a compassionate use program with teclistamab for RRMM after PI, IMiD, and CD38-antibody-exposure.

The patient was hospitalized and received multimodal supportive therapy for his general stabilization. Due to persisting bone pain, hypercalcemia, and renal impairment, a short-term bridging therapy with 200mg/day cyclophosphamide and dexamethasone 20mg/day for 5 days was given to limit disease progression until teclistamab initiation. Thereafter, step-up dosing of teclistamab was performed according to the recommended dosing schedule, and thereupon given at 1.5mg/kg/week in the outpatient setting (Fig. 1B). No signs of cytokine release syndrome (CRS) or immune effector cell-associated neurotoxicity syndrome (ICANS) occurred during initiation or thereafter [7, 8]. Upon hospital discharge, the general condition was substantially improved (ECOG 1, R-MCI 5/9). The patient achieved a complete remission (CR) with normalized λ-SFLCs of <0.5mg/L 1 and 2 months after teclistamab initiation (Fig. 1B, Suppl. Table 1). Mild hematologic adverse events, without need for supportive treatment, occurred (Common Terminology Criteria of Adverse Events [CTCAE] grade I pancytopenia) [9].

This case impressively demonstrates the feasibility, safety and efficacy of teclistamab treatment, also in very elderly patients (>80 years=octogenarians) suffering from symptomatic RRMM after PI, IMiD, and 38-antibody-exposure, suggesting that difficult-to-treat, elderly and/or frail patients may benefit from BCMA- or GPRC5D-bispecifics and others to come [10]. In line with this, we have now treated two other octogenarians with teclistamab in the same program, also achieving a rewarding response, good tolerance after as yet median follow-up of 7 weeks (range: 5–12) and all three patients continuing outpatient teclistamab treatment very successfully (Suppl. Table 1). With much longer follow-up, we thrive to report again on these very challenging cases as representatives of a difficult-to-treat patient cohort, that also greatly benefits from the fascinating innovations in modern oncology.

### Supplementary information

ESM 1 (DOCX 16.5 kb)
