# Supplementary material for: Safe and successful teclistamab treatment in very elderly multiple myeloma (MM) patients: a case report and experience from a total of three octogenarians
Source: Ann Hematol. 2023 Sep 15;102(12):3639–41. doi: 10.1007/s00277-023-05451-8 (PMC10640491; doi:10.1007/s00277-023-05451-8)
Supplement: Supplementary file 1 — (DOCX 16.5 kb) [file 277_2023_5451_MOESM1_ESM.docx]

**Supplementary Table 1. Detailed patient characteristics of octogenarians treated with the bispecific BCMA antibody teclistamab**

| **#** | **MM type** | **ID of MM** | **Prior # of anti-MM therapies** | **Age @ Tec start (y)** | **MM symptoms before Tec treatment** | **Tec tolerance (CRS / ICANs; CTC criteria)** | **In Tec cycle #** | **Treatment duration (weeks)** | **Ongoing out-pt Tec treatment** | **Response** | **R-MCI change before : on Tec treatment** |
| --- | --- | --- | --- | --- | --- | --- | --- | --- | --- | --- | --- |
| 1 | Lambda LC | 7/2021 | 3 | 82y | **CRAB**: 4/4 | ++: CRS: 0, ICANs: 0 | 11 | 12 | Yes | CR | **7**/9 🡪 **5**/9: frail 🡪 intermediate-fit |
| 2 | IgA kappa | 6/2018 | 4 | 87y | CR**AB**: 2/4 | ++: CRS: 2, ICANs: 0 | 6 | 7 | Yes | VGPR | **6**/9 🡪 **5**/9: intermediate-fit |
| 3 | Lambda LC | 11/2021 | 4 | 84y | CR**AB**: 2/4 | ++: CRS: 1, ICANs: 0 | 4 | 5 | Yes | VGPR | **4**/9 🡪 **2**/9: intermediate-fit 🡪 fit |
| ∑ | λ-LC: 2, IgAκ: 1 |  | In all: 3-4 | Mean: 84y | Symptomatic MM in all 3 | Excellent tolerance | Mean/Median follo-up:  8 weeks/7 weeks | | | CR: 1, VGPR: 2 | Comorbidity improvement via R-MCI in all 3 |

**Abbreveations**:

PIZ: pt-identity-#, MM: multiple myeloma, ID: initial diagnosis, #: number, @: at, Tec: teclistamab, y: years, CRS: cytokine release syndrome, ICANS: immune effector cell-associated neurotoxicity syndrome, CTC: common toxicity criteria, CR: complete response, VGPR: very good partical repsonse (according to IMWG [international myeloma working group] criteria), R-MCI: revised myeloma comorbidity index (www.myelomacomorbidityindex.org/en_calc.html), LC: light chain, CRAB. Hypercalcemia, renal impairment, anemia, bone lesions, out-pt: outpatient, ∑: summary of results
